# The effect of Latin dance on social physique anxiety in middle school girls: a pilot study

**DOI:** 10.3389/fpubh.2025.1564558

**Published:** 2025-06-03

**Authors:** Xutao Liu, Xiaozhuo Wei, Kim Geok Soh, Yingjie Lu, Rongzhi Li

**Affiliations:** ^1^School of Physical Education, Jiangsu University of Science and Technology, Zhenjiang, China; ^2^Department of Sports Studies, Faculty of Educational Studies, Universiti Putra Malaysia, Serdang, Malaysia; ^3^Department of Music, Faculty of Human Ecology, Universiti Putra Malaysia, Serdang, Malaysia; ^4^School of Physical Education, Shanghai University of Sport, Shanghai, China

**Keywords:** Latin dance, social physique anxiety, adolescents, intervention, mental health

## Abstract

**Introduction:**

Social physique anxiety (SPA) is a prevalent psychological issue among adolescents, particularly among female middle school students. SPA is characterized by fear of negative evaluation based on physical appearance and can significantly impact self-esteem and social interactions. This study aimed to investigate the effects of a 4-week Latin dance intervention on reducing SPA in middle school girls.

**Methods:**

A total of 40 female middle school students were randomly assigned to either the experimental group (*n* = 20) or the control group (*n* = 20). The experimental group participated in Latin dance training, consisting of two 40-minute sessions per week for four weeks. The control group engaged in traditional physical education classes, focusing on basketball. SPA was measured using the Social Physique Anxiety Scale (SPAS) before and after the intervention, assessing three dimensions: Negative Evaluation (NE), Self-performance (SP), and Social Comparison (SC).

**Results:**

The results indicated that the Latin dance intervention significantly reduced SPA across all three dimensions, with the most significant improvements observed in NE and SP (*p* < 0.05). The experimental group showed greater reductions in SPA compared to the control group (*p* = 0.004).

**Discussion:**

These findings suggest that Latin dance is an effective intervention for reducing SPA in adolescents. The improvements in SPA, particularly in NE and SP, highlight the potential of Latin dance to promote positive body image and enhance self-esteem. This study contributes to the growing body of research on the mental health benefits of dance and provides insights into integrating physical activity into psychological interventions aimed at improving adolescent well-being.

## 1 Introduction

Social physique anxiety (SPA) is a prevalent psychological issue among adolescents, particularly female middle school students, as they navigate significant physical and emotional changes during this period of life (1). SPA refers to the anxiety individuals experience in response to their body image, specifically regarding how they perceive others evaluating their physical appearance (2, 3). This form of anxiety can lead to negative psychological outcomes such as depression, lowered self-esteem, and social withdrawal (4, 5), all of which can significantly impact adolescents’ social interactions, emotional well-being, and overall quality of life (6, 7).

Adolescence is a critical developmental stage where individuals are particularly sensitive to their body image, with heightened concerns about appearance and the fear of negative judgment from others (8). These concerns can manifest as increased SPA, potentially leading to difficulties in socialization and overall mental health challenges. Studies indicate that up to 20% of adolescents experience some form of anxiety, with girls being particularly vulnerable to body image-related issues (9–11). SPA is often explained through the lens of self-presentation theory (12), which suggests that individuals experience anxiety when they believe others are judging their appearance in social contexts. Given the prevalence of SPA and its potential long-term impact on mental health, addressing this issue early is essential.

Previous research has demonstrated that physical activity, especially aerobic exercise, plays a significant role in reducing SPA (13–15). Aerobic exercises, which enhance both cardiovascular health and mental well-being, have been found to alleviate anxiety by promoting endorphin release and improving self-image (16). However, while physical exercise is well-recognized for its mental health benefits, studies focusing on the role of dance as a dynamic, rhythm-based physical activity in reducing SPA remain limited (17–19). Dance-based interventions not only incorporate physical movement but also encourage emotional expression and social bonding, which can be particularly effective for adolescents struggling with body image concerns (20). Dance, especially in group settings, combines physical exertion with social interaction, providing a holistic approach that may offer additional benefits compared to traditional forms of exercise (21).

Latin dance, in particular, offers a unique form of physical activity with its rhythmic movements, expressive nature, and social interaction (22, 23). Unlike traditional exercise or sports, Latin dance encourages self-expression and enhances body awareness through music and movement, which may significantly improve an individual’s relationship with their body and reduce SPA (24). Moreover, compared to more competitive or performance-based sports, Latin dance provides a non-threatening and inclusive environment where adolescent girls may feel less judged and more confident (17, 22). Given its engaging and inclusive nature, Latin dance is a promising intervention for addressing SPA, particularly among adolescents, and could be an accessible and enjoyable alternative to more conventional therapeutic approaches.

Despite the increasing use of physical activity to address adolescent mental health, few studies have specifically explored Latin dance as an intervention for SPA in female adolescents. This pilot study aims to explore the effects of a 4-week Latin dance intervention on SPA in female middle school students. By investigating both the physical and social components of Latin dance, this study seeks to evaluate whether it can effectively reduce SPA in this vulnerable population. Furthermore, this research contributes to the growing body of literature on dance as a therapeutic tool, with the potential to integrate it into educational or psychological interventions aimed at improving adolescents’ mental health and well-being.

## 2 Materials and methods

### 2.1 Participants

The study included 40 female middle school students, aged 12–15 years, who were recruited from a local school in Jiujiang City, Jiangxi Province, China. The participants were randomly assigned into two groups: the experimental group (*n* = 20) and the control group (*n* = 20). All participants were healthy, with no prior experience in Latin dance, and had no known physical or mental disorders. The Ethics Committee of the 171 Hospital in Jiujiang City, Jiangxi Province, China, approved the study (ethical approval number: 2022001). Written informed consent was obtained from both the participants and their parents before participation.

### 2.2 Study design

This study used a true experimental pretest-posttest control design to evaluate the effect of Latin dance on social physique anxiety (SPA) in middle school girls. The experimental group underwent a 4-week Latin dance intervention, which included two 40-min sessions per week. The control group participated in their usual physical education classes, which primarily focused on basketball. The study aimed to assess the effect of Latin dance compared to traditional physical activities on SPA levels.

The participants were randomly assigned to either the experimental or control group to avoid potential bias and contamination between groups. Random assignment also helped ensure that any observed differences could be attributed to the intervention.

### 2.3 Intervention

The experimental group participated in a 4-week Latin dance training program, which consisted of two 40-min sessions each week. The first 2 weeks focused on Cha-cha, followed by Rumba in the last 2 weeks. This choice of dances provided both aerobic exercise and social interaction through rhythmic, expressive movements.

The control group continued their regular physical education activities, which primarily involved basketball training. This was selected as the control activity because it involves physical exertion and social interaction, similar to the experimental group, allowing a comparison between traditional physical exercise and dance.

### 2.4 Procedure

The procedure followed a true experimental pretest-posttest design. Participants in both groups completed the pre-test on SPA levels prior to the intervention. The Social Physique Anxiety Scale (SPAS) questionnaires were administered in a quiet classroom setting by trained research assistants who were not involved in the intervention sessions, in order to reduce bias. The experimental group then participated in the 4-week Latin dance intervention, while the control group engaged in regular basketball activities.

Each intervention session (dance or basketball) was conducted by a certified instructor: the Latin dance sessions were led by a licensed dance teacher with over 5 years of experience teaching adolescents, and the school’s physical education teacher conducted the basketball sessions. Both instructors followed standardized session plans to ensure consistency across groups.

**Figure 1:**
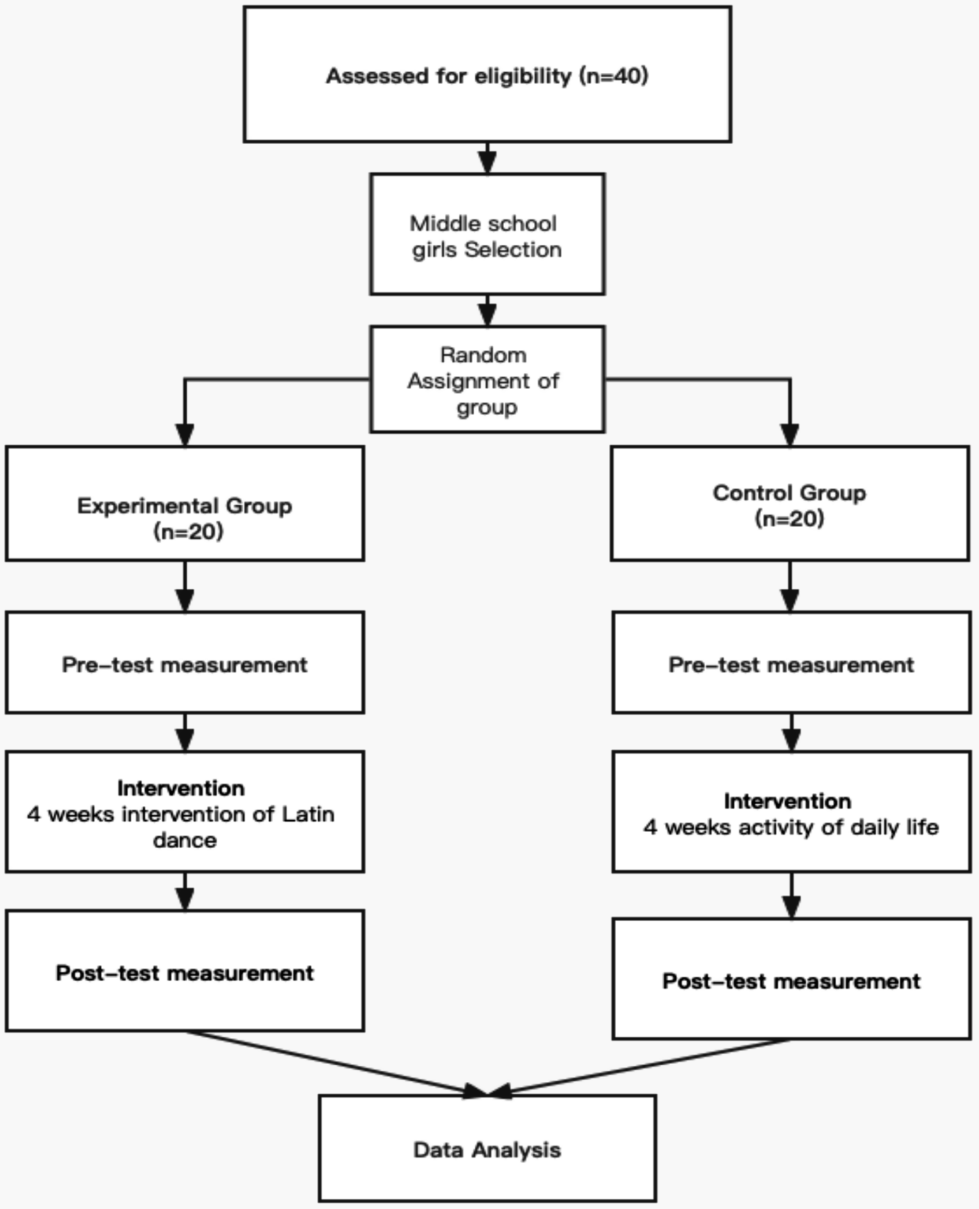
The specific pilot experiment process.

At the end of the intervention, both groups completed a post-test to assess any changes in SPA. The same research assistants administered the post-test using the same procedures and location as the pre-test, ensuring consistency in data collection. Figure 1 illustrates the specific pilot experiment process, detailing the timeline and key stages of the intervention, including the pre-test, intervention phase, and post-test.

### 2.5 Experimental controls

To minimize external influences on the study results, several experimental controls were implemented: Sample Selection: All participants were from the same school, ensuring that factors such as dietary habits and extracurricular activities were similar across groups. Additionally, participants were from different campuses to control for any environmental differences between the campuses. Pre-study Assessment: Participants in both the experimental and control groups underwent a pre-study physical fitness assessment to ensure comparability between groups in terms of baseline physical fitness. Consistency in Intervention: The teaching progress and the total intervention time for both groups were kept consistent. The experimental group underwent Latin dance training sessions twice per week for 4 weeks, while the control group followed the standard physical education basketball training regimen. Additionally, participants were informed that their participation in the study would not affect their academic performance or grades. This helped to create a relaxed and trustworthy environment where participants could complete the surveys without any concerns about their performance.

### 2.6 Data collection

Social physique anxiety (SPA) was assessed using the Social Physique Anxiety Scale (SPAS), a validated 12-item scale designed to measure anxiety related to body image. The scale assesses three dimensions of SPA: Negative Evaluation (NE), Self-performance (SP), and Social Comparison (SC). Participants rated each item on a 5-point Likert scale, ranging from 1 (“not at all”) to 5 (“extremely”). Higher scores indicate higher levels of anxiety. Participants completed the SPAS before the intervention (pre-test) and after the 4-week intervention (post-test). These data were used to assess changes in SPA due to the Latin dance intervention.

### 2.7 Data analysis

Data were analyzed using SPSS 25.0 software. Descriptive statistics, including means, standard deviations, frequencies, and percentage distributions, were computed for demographic and SPA-related data. To assess the effect of the 4-week Latin dance intervention on social physique anxiety (SPA), repeated measures analysis of variance (ANOVA) was used to examine the within-subject changes in SPA scores from pre-test to post-test for both the experimental and control groups. Repeated measures ANOVA is particularly appropriate for analyzing data with multiple measurements from the same subjects, as it accounts for the correlations between pre-test and post-test scores. This method allows us to evaluate changes over time within each group while considering the within-subject variability. Independent *t*-tests were also employed to compare the SPA scores between the experimental and control groups at both the pre-test and post-test stages. This comparison helps to determine whether the intervention led to significant differences between the two groups. A significance level of *p* < 0.05 was considered statistically significant. Effect sizes (Cohen’s *d*) were calculated to assess the magnitude of differences between groups at pre-test and post-test stages, providing additional context to the statistical significance by quantifying the strength of the intervention effect.

## 3 Results

### 3.1 Descriptive statistics

Descriptive statistics were computed to summarize the SPA scores of participants in both the experimental and control groups at pre-test and post-test. At the pre-test stage, the experimental group exhibited slightly higher mean scores across all SPA dimensions (NE: 13.95, SP: 17.70, SC: 7.80) compared to the control group (NE: 12.85, SP: 16.40, SC: 7.90). This indicates that participants in the experimental group reported marginally higher levels of social physique anxiety before the intervention. After the intervention, the experimental group displayed notable reductions in SPA scores, with the mean total SPA score decreasing from 39.45 (pre-test) to 32.25 (post-test). These reductions were observed consistently across all three dimensions, suggesting an overall improvement in their social physique anxiety levels. In contrast, the control group did not show significant changes in total SPA scores between pre-test (37.15) and post-test (37.80), and minor fluctuations in the dimensions were noted. For instance, while SC showed a slight decrease from 7.90 to 7.35, NE increased from 12.85 to 15.05, indicating no consistent trend of improvement. Table 1 provides a detailed summary of the descriptive statistics for SPA scores at pre-test and post-test for both groups.

**Table 1:** Descriptive statistics for SPA scores in the experimental and control groups at pre-test and post-test.

Group	Time point	NE (Mean ± SD)	SP (Mean ± SD)	SC (Mean ± SD)	SPA (Mean ± SD)
Experimental group	Pre-test	13.95 ± 2.87	17.70 ± 2.79	7.80 ± 1.70	39.45 ± 6.49
	Post-test	12.40 ± 2.87	13.45 ± 3.46	6.40 ± 1.50	32.25 ± 5.97
Control group	Pre-test	12.85 ± 4.00	16.40 ± 1.82	7.90 ± 1.74	37.15 ± 5.93
	Post-test	15.05 ± 2.95	15.40 ± 2.50	7.35 ± 1.53	37.80 ± 5.51

### 3.2 Within-group changes

Repeated measures analysis of variance (ANOVA) revealed significant within-group changes in SPA scores in the experimental group from pre-test to post-test (*F* = 8.07, *p* = 0.007). Notably, all three dimensions of SPA—NE, SP, and SC—exhibited statistically significant improvements (*p* < 0.05). The paired sample *t*-test further confirmed these changes, with significant reductions observed in NE (*t* = 2.142, *p* = 0.045), SP (*t* = 4.044, *p* = 0.001), and SC (*t* = 4.273, *p* < 0.001). The overall SPA score also demonstrated a marked decrease (*t* = 4.481, *p* < 0.001). These results suggest that the intervention of Latin dance effectively reduced SPA within the experimental group. In contrast, the control group did not experience significant changes in SPA scores between the pre-test and post-test (*F* = 2.08, *p* > 0.05), indicating that basketball training had a limited impact on SPA. Table 2 presents the paired sample *t*-test results for each SPA dimension.

**Table 2:** Paired sample *t*-test results for within-group changes in SPA scores.

Dimension	Pre-test Mean ± SD	Post-test Mean ± SD	*t*-value	*p*-value
Negative evaluation	13.95 ± 2.87	12.40 ± 2.87	2.142	0.045
Self-performance	17.70 ± 2.79	13.45 ± 3.46	4.044	0.001
Social comparison	7.80 ± 1.70	6.40 ± 1.50	4.273	0.000
SPA (Total score)	39.45 ± 6.49	32.25 ± 5.97	4.481	0.000

### 3.3 Between-group differences

To evaluate the effectiveness of Latin dance compared to basketball training, independent *t*-tests were conducted to analyze the between-group differences in SPA scores and their dimensions at the post-test. As shown in Table 3, the experimental group demonstrated significantly lower scores in SPA dimensions than the control group after the intervention. In the Negative Evaluation (NE) dimension, the experimental group scored significantly lower (Mean = 12.40, SD = 2.87) compared to the control group (Mean = 15.05, SD = 2.95), with a *t*-value of −2.880 and *p* = 0.007, indicating a statistically significant difference. For the Self-performance (SP) dimension, the experimental group (Mean = 13.45, SD = 3.46) also showed significantly lower scores than the control group (Mean = 15.40, SD = 2.50), with a *t*-value of −2.044 and *p* = 0.048. However, in the Social Comparison (SC) dimension, the difference between the experimental group (Mean = 6.40, SD = 1.50) and the control group (Mean = 7.35, SD = 1.53) did not reach statistical significance, with a *t*-value of −1.981 and *p* = 0.055. For the overall Social Physique Anxiety (SPA) score, the experimental group achieved a significantly greater reduction (Mean = 32.25, SD = 5.97) compared to the control group (Mean = 37.80, SD = 5.51), with a *t*-value of −3.054 and *p* = 0.004. These findings suggest that Latin dance was more effective than basketball training in reducing overall SPA, particularly in the Negative Evaluation and Self-performance dimensions. While the Social Comparison dimension also showed a trend toward improvement, it did not achieve statistical significance.

**Table 3:** Comparison of social physique anxiety and its dimensions between the experimental and control groups after intervention.

Dimension	Group	Post-test Mean ± SD	*t*-value	*p*-value
Negative evaluation (NE)	Experimental	12.40 ± 2.87	−2.880	0.007
	Control	15.05 ± 2.95		
Self-performance (SP)	Experimental	13.45 ± 3.46	−2.044	0.048
	Control	15.40 ± 2.50		
Social comparison (SC)	Experimental	6.40 ± 1.50	−1.981	0.055
	Control	7.35 ± 1.53		
SPA (Total score)	Experimental	32.25 ± 5.97	−3.054	0.004
	Control	37.80 ± 5.51		

## 4 Discussion

### 4.1 Summary of findings

The results of this pilot study support the hypothesis that Latin dance can reduce social physique anxiety (SPA) in female middle school students. Specifically, significant reductions in SPA scores were observed in the experimental group, particularly in the dimensions of Negative Evaluation (NE) and Self-performance (SP). These findings align with previous research indicating that physical activity, including dance, can improve body image and reduce anxiety related to physical appearance (25, 26). The control group, which participated in basketball training, did not show significant changes in SPA scores, suggesting that the unique aspects of Latin dance, such as its rhythmic movements and social interaction, contributed to the observed improvements in SPA.

### 4.2 Interpretation of results

The significant reductions in SPA observed in the experimental group can be attributed to several factors associated with Latin dance. First, the expressive nature of Latin dance encourages self-expression, which may help participants develop a more positive relationship with their bodies. Second, the rhythmic and music-based elements of Latin dance likely contributed to mood enhancement and anxiety reduction, consistent with research showing that music and movement can alleviate anxiety and improve psychological well-being (27, 28). Additionally, the social aspect of group dance, where participants engage with others in a supportive environment, may have further reduced social physique anxiety by minimizing fears of judgment.

The lack of significant changes in the control group suggests that traditional physical activities like basketball, although beneficial for general physical health, may not be as effective in addressing the psychological aspects of SPA, such as body image concerns. This highlights the potential of dance as a more holistic intervention for addressing SPA in adolescents, especially when compared to other physical activities.

### 4.3 Implications for practice

The findings of this study have several practical implications. First, Latin dance can be considered a promising intervention for improving body image and reducing SPA among middle school girls. Schools and therapists could incorporate Latin dance into physical education programs or therapeutic settings to promote mental health and well-being. Additionally, as a non-competitive, socially engaging activity, Latin dance may be particularly suitable for adolescents who may feel self-conscious or excluded in traditional sports settings.

### 4.4 Study limitations

This study has several limitations. First, the sample size was relatively small (*n* = 40), which may limit the generalizability of the findings. As a pilot study, its primary aim was to explore preliminary effects and feasibility; however, larger sample sizes are required in future studies to enhance statistical power and enable subgroup analysis (e.g., based on SPA dimensions or baseline anxiety levels).

Second, the study only measured SPA as an outcome, and other psychological variables, such as self-esteem or depression, were not assessed. Future research could explore the broader psychological benefits of Latin dance and examine whether it has lasting effects beyond the 4-week intervention.

Third, the intervention period of 4 weeks is relatively short. Social physique anxiety is considered a stable psychological trait, and short-term improvements may not reflect enduring changes. The limited duration makes it uncertain whether the observed effects can be maintained over time, thus necessitating longitudinal studies with extended interventions and follow-up assessments to evaluate long-term efficacy.

Additionally, the study relied on self-reported measures of SPA, which may be subject to bias. Future studies could use more objective measures, such as physiological indicators of stress or anxiety, to complement the self-report data.

### 4.5 Future directions

Future research should focus on replicating this study with a larger and more diverse sample to strengthen the findings. Including participants from different regions, cultural backgrounds, and educational environments will help improve the generalizability and applicability of the results to broader adolescent populations.

Furthermore, longitudinal studies could assess the long-term effects of Latin dance on SPA and other aspects of adolescent mental health. Extending the intervention duration and conducting follow-up assessments at multiple time points will be crucial to evaluate whether the intervention’s positive effects are sustained over time.

Exploring the impact of different dance styles or incorporating qualitative data could provide deeper insights into the mechanisms behind the reduction in SPA. Future studies should consider mediation analysis, qualitative interviews, or mixed methods approaches to investigate the specific roles of self-expression, musical engagement, and social interaction in the intervention’s effectiveness.

Finally, investigating the role of cultural factors in the effectiveness of dance interventions could help tailor programs to specific populations. Applied research in real-world educational and clinical settings, along with the development of standardized intervention protocols and practical implementation guidelines, will be essential for enhancing the feasibility and scalability of such interventions.

## 5 Conclusion

This pilot study demonstrated that a 4-week Latin dance intervention effectively reduced social physique anxiety (SPA) in female middle school students, particularly in the dimensions of Negative Evaluation (NE) and Self-performance (SP). These findings suggest that Latin dance, with its combination of physical activity, rhythmic movement, and social interaction, can be a valuable tool for improving body image and reducing anxiety in adolescents. While the study’s small sample size and short duration are limitations, the results provide a strong foundation for further research into the long-term effects of dance interventions on adolescent mental health. Future studies should involve larger samples and explore additional psychological outcomes to confirm and expand on these findings. In conclusion, Latin dance is a promising, accessible intervention for reducing SPA in middle school girls, with potential applications in both educational and therapeutic settings.

## Data Availability

The original contributions presented in the study are included in the article/supplementary material, further inquiries can be directed to the corresponding authors.
